# Frequency, Risk Factors, and Outcome of Definite Stent Thrombosis: A Single-Center Experience

**DOI:** 10.7759/cureus.27240

**Published:** 2022-07-25

**Authors:** Ashique Ali Khoso, Ghulam H Soomro, Sarwan B Mal, Rehan Malik, Bashir Hanif, Rozi Khan

**Affiliations:** 1 Cardiovascular Medicine, Pir Abdul Qadir Shah Jeelani (PAQSJ) Institute of Medical Sciences, Gambat, PAK; 2 Cardiology, Tabba Heart Institute, Karachi, PAK; 3 Interventional Cardiology, Ziauddin University, Karachi, PAK; 4 Cardiology, National Institute of Cardiovascular Diseases (NICVD), Karachi, PAK; 5 Clinical Research, Tabba Heart Institute, Karachi, PAK; 6 Internal Medicine, Medical University of South Carolina, Charleston, USA; 7 Internal Medicine, Bolan University of Medical and Health Sciences, Quetta, PAK

**Keywords:** subacute stent thrombosis, acute stent thrombosis, extremely late stent thrombosis, late stent thrombosis, bare metal stents (bms), primary percutaneous coronary intervention (pci), very late stent thrombosis, drug eluting stents (des), coronary stent thrombosis

## Abstract

Introduction

Stent thrombosis (ST) is a serious and potentially life-threatening complication of primary or complex high-risk percutaneous coronary intervention (PCI). Multiple factors are said to precipitate ST, related to the patient's clinical comorbidities, lesion characteristics, operative technique, and post-procedural care. The older-generation stents were thought to be involved in early ST. Though the new generation of drug-eluting stents decreases the incidence of early and late ST, patients are still at risk of very late stent thrombosis (VLST).

Objective

To evaluate the frequency, risk factors, and outcomes of definite ST in developing and resource-constrained countries like Pakistan, where PCIs, including primary PCI, complex PCI, and PCI in high-risk populations, are performed routinely.

Methods

This observational cross-sectional study included all patients who underwent primary and complex high PCI between 2012 and 2017 at TABBA Heart Institute (THI), Karachi, Pakistan.

Results

We included a total of 6587 patients in our study, and among the enrolled sample size, 22 (0.33%) had definite ST. Acute stent thrombosis (AST) was found in seven patients, sub-acute stent thrombosis (SAST) in 10, late stent thrombosis (LST) in two, and VLST were observed in three patients. The basic characteristics of our study ST population were as follows: mean age was 58 years, 95.5% were male, 4.5% were female, nine patients (40%) had diabetes mellitus, 15 patients (68%) had hypertension, 11 (50%) had dyslipidemia, and four patients were smokers (18%).

Conclusion

The frequency, risk factors, and rate of mortality of definite ST in the Pakistani population who underwent primary and complex high-risk PCI reflect nearly equal statistics observed in other studies. As seen in other international studies, the incidence rate of VLST was higher in our population.

## Introduction

Stent thrombosis (ST) is a life-threatening complication of any percutaneous coronary intervention (PCI), resulting from total or near-total occlusion of the stent lumen by thrombus, and this complication can happen anywhere from the intraprocedural stage to years after the stent deployment [1]. ST is an uncommon but serious complication that carries significant mortality and morbidity [1].

With the innovation of the bare-metal stent (BMS), a much lower incidence of restenosis and acute occlusion was observed, compared to balloon angioplasty. With the increased use of PCI, the primary focus was more advanced from procedural success to the prevention of in-stent thrombosis. Later, the invention of drug-eluting stents (DES) in clinical practice was initially thought to be the solution to reduce the restenosis rate in BMS. Still, effervescence was alleviated by DES' association with late stent thrombosis (LST) and very late stent thrombosis (VLST) [1].

Despite considerable improvements in antiplatelet therapy, thrombotic events remain the major cause of death after PCI [2,3]. As ST is one of the dangerous complications of PCI, its incidence and prevalence are being extensively studied and observed closely. The incidence of ST followed a linear line with the development of contemporary, improved stents and antiplatelets. Although there are immense data to support the clinical trials on ST, there is much disagreement about whether the randomized controlled trials truly reflect “real-world” data applicable in clinical practice [1].

Most STs occur within the first 30 days after PCI. In general, in clinical practice, the anticipated rate of early ST is ∼1%, and beyond 30 days, 0.2%-0.6% per year [4]. First-generation DES and LST occur steadily at an annual rate of 0.4%-0.6% for up to four years [5]. The incidence of LST and VLST in patients with BMS has been imperfectly characterized. A retrospective analysis of 4,503 patients treated with at least one BMS revealed the summative incidence of ST at 10 years to be 2% [6].

The incidence of explicit or likely ST also varies by the type of stent used. In a meta-analysis of randomized controlled trials of DES [7], the incidence was 0.1% vs 1.0% in the sirolimus-eluting stent group and 0.4% vs. 0.3% in the paclitaxel-eluting stent group, respectively, compared to the corresponding BMS group. Most perilously, and surprisingly, acute ST has been observed with mortality rates of 20%-45% [8] and myocardial infarction (MI) rates of 50%-70% [8]. Further, roughly 20% of the patients who have ST have a recurrent episode within two years [9].

The rationale behind this study is to evaluate the frequency, risk factors, and outcome of ST in developing and resource-constrained countries like Pakistan, where PCIs including primary PCI, complex PCI, and PCI in high-risk populations are performed routinely.

## Materials and methods

Study design and setting

This cross-sectional study was conducted at one of the busiest cardiac hospitals in Pakistan between 2012 and 2017. The study was approved by the Institutional Review Board (THI/IRB/21/2016).

Patient selection

All patients who had undergone PCI at the center during the study period were included in our study, with the exclusion of all those whose PCI was done outside the primary study center and patients who developed in-stent restenosis. All patients were managed as per standard institutional protocol. All PCIs were conducted by a consultant interventional cardiologist with a minimum of six years of experience in interventional cardiology.

Data collection and statistical analysis

Data for the study were obtained in a structured proforma consisting of demographic, clinical, and procedural characteristics, and outcomes.

The definition, classification, and timing of ST were categorized as proposed by the Academic Research Consortium, which is based on the timing of events.

-acute stent thrombosis (AST) - 0-24 hours after stent implantation;

-subacute stent thrombosis (SAST) - 24 hours to 30 days after stent implantation;

-LST - 30 days to one year after stent implantation;

-VLST - one year after stent implantation.

Data were exhibited as mean ± standard deviation or percentages. Pearson's chi-square test for categorical/qualitative measurement and student's t-test for continuous parameters were used to compare the different groups. Multiple logistic regression analyses were employed to explore factors, including the demographic, clinical, and revascularization characteristics, related to the outcomes of patients after revascularization. All tests of significance are two-tailed and p <0.05 was considered significant. All statistical analyses were performed using IBM SPSS Statistics 19 software (Chicago, IL, USA).

## Results

**Figure 1:**
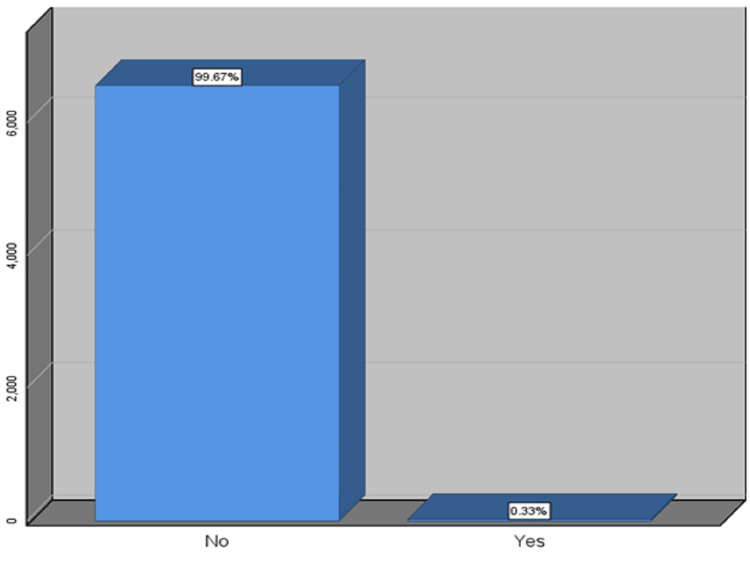
Percentage distribution of the study population affected by stent thrombosis.

During the study period, a total of 6587 patients were included in the study, of whom 22 (0.337%) patients presented with ST (Figure 1). Among the patients who had ST, the mean age was 58.8 years, 21 patients (95%) were male, 4.5% female, nine patients (41%) had diabetes mellitus (DM), 15 (68%) had hypertension (HTN), 11 (50%) had dyslipidemia, and four patients were smokers (18%). The multivessel disease was seen more often (59%) than the single-vessel disease (41%). The baseline and periprocedural characteristics of the study population are depicted in Table 1.

**Table 1:** Baseline and periprocedural basic characteristics of the study population. MI: myocardial infarction; CABG: coronary artery bypass graft; NSTEMI: non-ST elevation myocardial infarction; STEMI: ST elevation myocardial infarction; PCI: percutaneous coronary intervention; LVEF: left ventricular ejection fraction; TIMI: thrombolysis in myocardial infarction; SD: standard deviation; BMS: bare-metal stent; DES: drug-eluting stent.

Characteristics	Overall N = 6587	Cumulative Stent Thrombosis N = 22	
Male sex, No. (%)	5430 (82.7%)	21 (95.5%)	
Female sex, No. (%)	1135 (17.2%)	01 (4.5%)	
Age, mean (SD), years	56.81 (10.9)	58.86 (9.9)	
Clinical characteristics			
Diabetes mellitus	2722 (41.5)	9 (41)	
Hypertension	3948 (60)	15 (68)	
Dyslipidemia	1909 (29)	11 (50)	
Current smoker	1712 (26)	4 (18)	
Prior MI	1121 (17)	10 (45.5)	
Prior PCI	646 (10)	14 (37)	
Prior CABG	375 (5.7)	0 (0)	
NSTEMI	1770 (27)	2 (9)	
STEMI	2487 (38)	16 (73)	
Dialysis	28 (0.4)	0(0)	
Single-vessel disease	2194 (33)	9 (41)	
Multivessel disease	4371 (67)	13 (59)	
Cardiogenic shock	205 (3)	2 (9)	
Pre PCI LVEF mean (SD), %	45 (11)	42 (12)	
Lesion characteristics			
Bifurcation	1121 (17)	6 (27)	
B2 or C type	3072 (47)	9 (41)	
Pre-intervention TIMI 3 flow	1112 (17)	2 (9)	
Thrombus	1910 (29)	20 (91)	
Procedural characteristics			
Stent length per lesion, mm (SD) DES and BMS	22 (11.5)	18 (8.8)	
Post-procedural TIMI flow			
TIMI 1 flow	30 (0.5)	0 (0)	
TIMI 2 flow	28 (0.4)	0 (0)	
TIMI 3 flow	116 (1.8)	1 (4.5)	
Perforation	28 (0.4)	0 (0)	
Significant dissection	312 (6514)	1 (21)	

The frequencies of patients presenting with ST-segment elevation myocardial infarction (STEMI), non-ST-segment elevation myocardial infarction (NSTEMI), single-vessel disease, multivessel disease, pre-PCI left ventricular ejection fraction, and cardiogenic shock are presented in Table 1. In our study, ST was significantly associated with age, HTN, dyslipidemia, DM, and smoking. It is equally important to highlight, however, that in our study, patients presenting with STEMI (73%) had a higher incidence of ST than NSTEMI (9%). Two (9%) patients in our study reported the premature discontinuation of dual antiplatelet therapy (DAPT).

A total of 6587 had PCI and stents inserted, and out of the total study population of 22 patients, 0.337% developed ST as shown in Figure 1.

**Figure 2:**
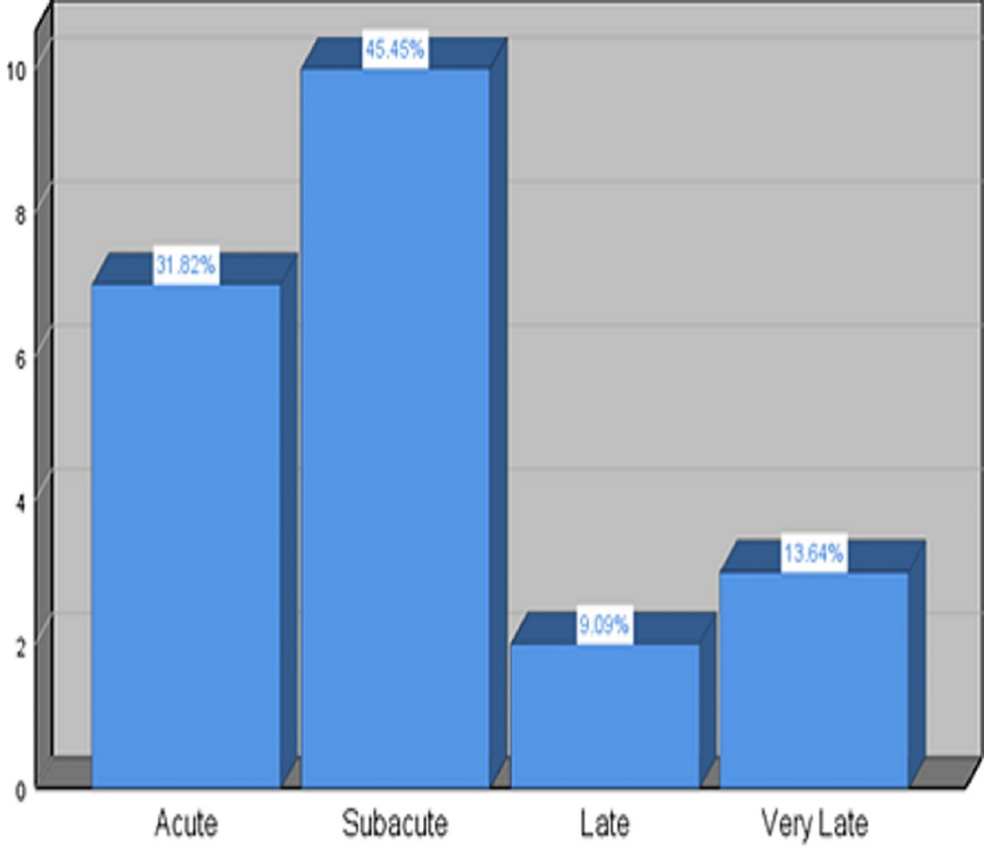
Frequencies of AST, SAST, LST, and VLST. AST: acute stent thrombosis: SAST: sub-acute stent thrombosis; LST: late stent thrombosis; VLST: very late stent thrombosis.

Acute stent thrombosis (AST) was found in seven patients (31.82%), with sub-acute stent thrombosis (SAST) being comparatively higher in our study and seen in 10 patients (45.45%), LST in two patients (9%), and VLST in three patients (13.64%), as seen in Figure 2. 

**Figure 3:**
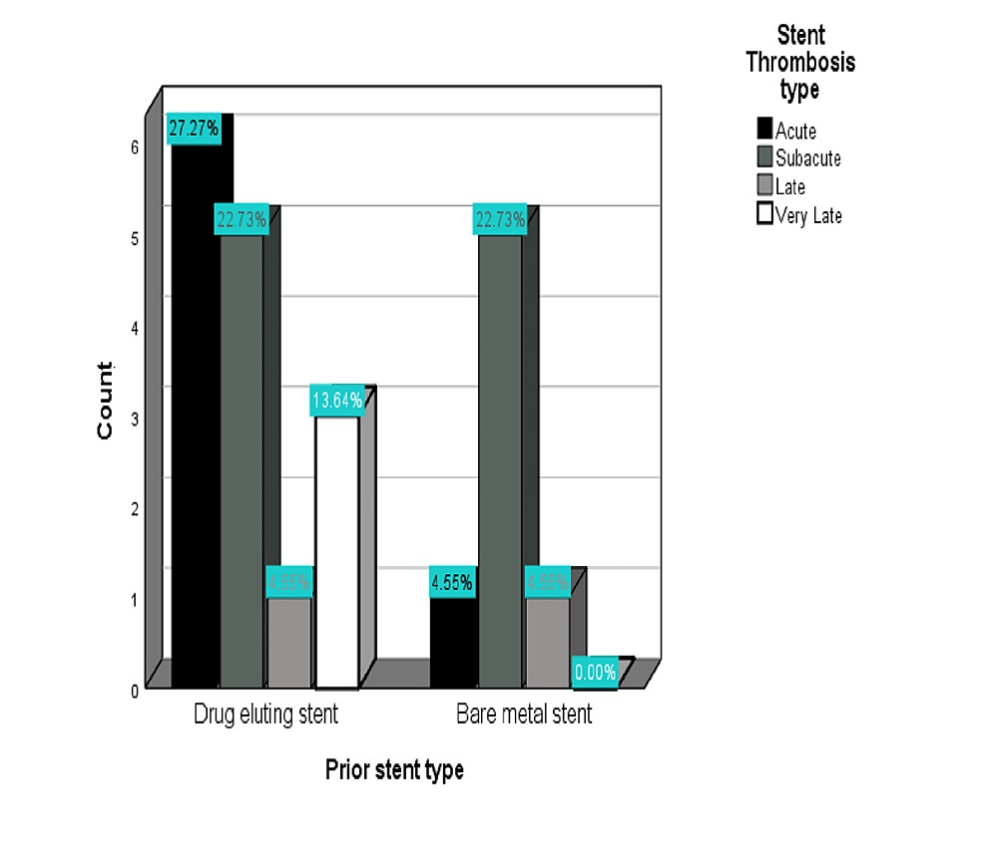
Percentage of stent thrombosis in DES and BMS sub-population. DES: drug-eluting stent; BMS: bare-metal stent.

The incidence of AST was higher (27.27%) in DES and marginally lower but significant (22.73%) in BMS. SAST and LST were almost equal in both DES and BMS at 27% and 4.5%, respectively, but VLST was common in DES at 13.64%, while none was observed in BMS, as reported in Figure 3. 

**Figure 4:**
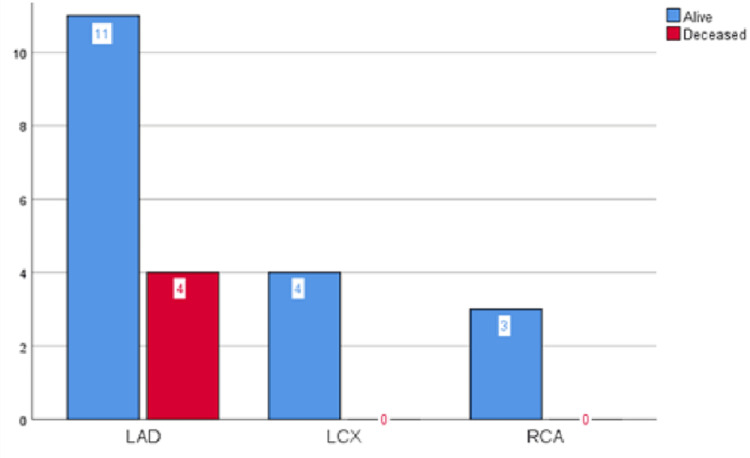
Outcome of stent thrombosis and its coronary artery distribution LAD: left anterior descending artery; LCX: left circumflex artery; RCA: right coronary artery.

Regarding the coronary anatomy, the left anterior descending artery (LAD) ST was observed in 15 (68%) patients, left circumflex artery (LCx) ST in four (18%), and right coronary artery (RCA) ST in three (14%) patients. Zero lesions were seen in the saphenous vein and arterial graft. Four (18%) patients out of 22 with definite ST died, all due to LAD ST. Notably, none of the deaths was reported in the LCx and RCA ST (Figure 4).

## Discussion

Our research was conducted in one of the busiest heart hospitals in Pakistan from 2012 to 2017. It aimed to evaluate the frequency, determinants, and outcomes of definite ST in all kinds of PCIs, including primary, complex, and high-risk groups. To our knowledge, ours is the first study locally to evaluate the importance of definite ST in all kinds of PCI. The frequency of definite ST in our study was 0.3%, which closely matches the international study findings reported above; but our study included PCI for both acute coronary syndrome (ACS) and stable patients.

ST is multifactorial; several risk factors are co-related and involved in the development of in-stent thrombus formation and its propagation. With the occurrence of ST, the cumulative risk of early ST in patients with ACS results from an agglomeration of various prothrombotic conditions such as the low-flow state in coronaries, the setting of distal embolization, poor stent apposition, local thrombus burden, an ulcerative plaque with necrotic components, plaque prolapse into stent lumen, underlying medical comorbidities that increase prothrombotic state, low ejection fraction, and improper use of antiplatelets [10].

Tariq et al. [11] studied the frequency and predictors associated with acute and sub-acute ST in patients who underwent only primary PCI, finding only three patients with definite ST, and the rest of the 30 patients with probable ST. The contemporary use of DAPT and high-pressure inflation has remarkably reduced the incidence of ST to 0.7% in one year and about 0.2%-0.6% in the next year. The incidence was even less observed for elective PCI (0.3%-0.5%) but was as high as 3.4% for ACS [12,13].

Table 2 explains the risk of early ST by type of stent and clinical presentation. As seen in the table, early ST with DES is determined mainly by patients' clinical presentation, with a low risk of ST in patients with stable coronary artery disease (CAD), moderate in patients diagnosed with unstable angina (UA)/NSTEMI and high risk in those who presented with acute STEMI [10].

**Table 2:** Risk of early stent thrombosis by type of stent and clinical presentation. STEMI: ST-segment elevation myocardial infarction; NSTEMI: non-ST-segment elevation myocardial infarction; UA: unstable angina.

	Stable Angina	UA/NSTEMI	STEMI
Bare-metal stents, %	0-0.5	1.4-1.6	0-2.9
Drug-eluting stents, %	0.3-0.4	1.2-1.9	0-3.1

Premature discontinuation of DAPT is the strongest predictor of early ST [14]. In our study, two patients discontinued DAPT and suffered definite ST. DM is another significant risk factor associated with definite ST [14,15]. In our study, 41% of the patients with definite ST were diabetic. Risk factors like male gender, HTN, multivessel disease, bifurcation, and complex lesions were almost similar in our study. Abnormal placement of stent struts and faulty fractured stents alter the stent integrity and in-stent flow and all these, in combination or alone, affect blood viscosity [16] and promote ST. In addition, all these factors that compromise the blood flow inside the stent can be established with the help of intra-coronary imaging techniques.

In our study, intravascular-ultrasound-proven under-expansion and stent edge dissection were found in two and five patients, respectively.

VLST was higher in DES, compared to BMS. However, anecdotal reports of VLST [17] were followed in the spring of 2006 by registry reports, raising the consternation that although DES lessens restenosis compared to BMS, an increase in rare but catastrophic late thrombotic complications might counterpoise such early transitive [18-20].

ST is observed to be the most dreadful complication of any successful PCI. Punctilious care during the stent deployment, good patient education to adhere and to tolerate DAPT, modifying risk factors, and regular follow-ups are integral strategies to prevent or at least minimize ST. Novel stents are emerging with the prospect of innately lessening the risk of ST. Any elective surgery after stent implantation (six weeks after BMS, 6-12 months after DES) should be postponed without stopping or holding of DAPT (if possible) and, finally, a more potent antiplatelet like ticagrelor should be used in a patient with ACS [11].

Limitation 

The results of this study are based on the experience of a single center. We cannot be certain about the comprehensive use of the probable determinants of ST in multivariate analysis. Further, multicenter studies with a larger number of patients need to be conducted in this part of the world to find definite incidence, determinants, and outcomes of obvious ST.

## Conclusions

In conclusion, our study has illustrated the frequency, risk factors, and rate of mortality of definite ST in the Pakistani population who underwent primary and complex high-risk PCI. Our study conclusions revealed data matching those of international studies. Almost half the patients were diabetic and there was increasing concern that VLST is slightly higher in newer DES. It is also important to note that ST was more commonly seen in an unstable presentation like UA/NSTEMI and STEMI than in stable CAD.
